# NRF2 Is an Upstream Regulator of MYC-Mediated Osteoclastogenesis and Pathological Bone Erosion

**DOI:** 10.3390/cells9092133

**Published:** 2020-09-21

**Authors:** Peter Sang Uk Park, Se Hwan Mun, Steven L. Zeng, Haemin Kim, Seyeon Bae, Kyung-Hyun Park-Min

**Affiliations:** 1 Arthritis and Tissue Degeneration Program, David Z. Rosensweig Genomics Research Center, Hospital for Special Surgery, New York, NY 10021, USA; sp727@cornell.edu (P.S.U.P.); Muns@hss.edu (S.H.M.); slz9@duke.edu (S.L.Z.); Kimha@hss.edu (H.K.); 2 Department of Medicine, Weill Cornell Medical College, New York, NY 10021, USA; 3 BCMB Allied Program, Weill Cornell Graduate School of Medical Sciences, New York, NY 10021, USA

**Keywords:** osteoclasts, MYC, NRF2, RANKL signaling

## Abstract

Osteoclasts are the sole bone-resorbing cells that play an essential role in homeostatic bone remodeling and pathogenic bone destruction such as inflammatory arthritis. Pharmacologically targeting osteoclasts has been a promising approach to alleviating bone disease, but there remains room for improvement in mitigating drug side effects and enhancing cell specificity. Recently, we demonstrated the crucial role of MYC and its downstream effectors in driving osteoclast differentiation. Despite these advances, upstream regulators of MYC have not been well defined. In this study, we identify nuclear factor erythroid 2-related factor 2 (NRF2), a transcription factor known to regulate the expression of phase II antioxidant enzymes, as a novel upstream regulator of MYC. NRF2 negatively regulates receptor activator of nuclear factor-κB ligand (RANKL)-induced osteoclastogenesis through the ERK and p38 signaling-mediated suppression of MYC transcription. Furthermore, the ablation of MYC in osteoclasts reverses the enhanced osteoclast differentiation and activity in NRF2 deficiency in vivo and in vitro in addition to protecting NRF2-deficient mice from pathological bone loss in a murine model of inflammatory arthritis. Our findings indicate that this novel NRF2-MYC axis could be instrumental for the fine-tuning of osteoclast formation and provides additional ways in which osteoclasts could be therapeutically targeted to prevent pathological bone erosion.

## 1. Introduction

In prevalent bone diseases such as rheumatoid arthritis and osteoporosis, overly-activated osteoclasts are the primary culprits of excessive bone loss and erosion [1,2]. Osteoclasts are multinucleated, bone-resorbing cells differentiated from myeloid lineage precursor cells with the stimulus from macrophage colony-stimulating factor (M-CSF) and receptor activator of nuclear factor-κB ligand (RANKL) [3,4,5,6,7]. Upon binding to their receptors, M-CSF and RANKL induce mitogen-activated protein kinases (MAPKs) such as extracellular signal-regulated protein kinase (ERK) and p38 to activate pathways in the proliferation and differentiation of osteoclast precursor cells [8]. Downstream of these pathways lies the master transcriptional factor nuclear factor of activated T cells (NFATc1), which promotes the transcription of essential genes for osteoclast function in bone resorption and polarization [9,10]. When these multinucleated osteoclasts resorb bone at a higher intensity than bone-forming osteoblasts, pathological bone loss occurs [11,12].

Pharmacological inhibitors of osteoclasts and bone resorption such as bisphosphonates have been used to treat pathological bone losses [13]. However, their side effects due to complex and undetermined mechanisms [14] have necessitated the development of more osteoclast-specific inhibitors with determined mechanisms such as denosumab, which inhibits the RANKL-RANK inhibition [15]. As an effort to further uncover the mechanisms behind osteoclastogenesis that could be therapeutically targeted, we recently revealed the critical role of MYC in osteoclast formation and metabolic reprogramming in osteoclasts [16,17]. Furthermore, MYC-deficient mice exhibited the osteopetrotic phenotype due to decreased osteoclast formation, and MYC positively regulated osteoclastogenesis by inducing the expression of NFATc1 and estrogen receptor-related receptor alpha (ERRα), which is the key governing factor of metabolic reprogramming [16]. MYC expression is also tightly regulated during osteoclastogenesis [16]. However, upstream regulators of MYC during osteoclastogenesis have not been determined yet [18].

Nuclear factor-erythroid 2-related factor 2 (NRF2; encoded by *NFE2L2*) is a basic region-leucine zipper (bZip) transcriptional factor known to regulate adaptive, cytoprotective responses [19,20]. NRF2 is sequestered in the cytoplasm by Kelch-like ECH-associated protein (KEAP1), which acts as an electrophile sensor for electrophiles and redox molecules [21]. Upon stress responses, NRF2 dissociates from KEAP1 and translocates into the nucleus. NRF2 then regulates the expression of genes that are involved in the response to cellular responses, such as antioxidant metabolism, detoxification, iron catabolism, and intermediate metabolism of carbohydrate and lipid, and facilitates the adaptation of cells to oxidative and electrophilic cellular stress signals. NRF2 is expressed in bone cells and plays an important role in bone homeostasis [22]. NRF2-deficient mice have a significantly lower bone mass and bone strength compared to wild-type (WT) mice [23,24]. NRF2 suppresses osteoclast differentiation, while the role of NRF2 in osteoblasts is more complicated [25,26,27]. ERK and p38 MAPKs are phosphorylated early on upon RANKL stimulation to propagate cell proliferation and differentiation signals [28,29]. Cross-regulation between ERK and p38 activation and NRF2 has been reported in bone cells and other cell types [25,30,31,32]. However, the exact mechanism by which NRF2 deficiency enhances osteoclastogenesis has not been fully elucidated.

In this study, we demonstrate that NRF2 acts as a novel upstream regulator of MYC by controlling the activation of ERK and p38 during RANKL-induced osteoclastogenesis. Specifically, NRF2 suppresses MYC expression by attenuating the RANKL-induced activation of ERK and p38, which is crucial for MYC induction. Meanwhile, knocking out MYC nullified the osteoclast-promoting effect of NRF2 deficiency both in vivo and in vitro, and protected NRF2-deficient mice from pathological bone loss during inflammatory arthritis, highlighting the significance of the NRF2-MYC axis in the regulation of osteoclastogenesis. Our findings indicate that this novel NRF2-MYC axis is crucial for the fine-tuning of osteoclastogenesis and provides strategies for which molecules could be therapeutically targeted to prevent pathological bone erosion.

## 2. Materials and Methods

### 2.1. Mice

All animal experiments were approved by the Institutional Animal Care and Use Committee (IACUC) of Weill Cornell Medical College (2015-062 and 2015-065, approved 12 April 2018). Animals were housed in a specific pathogen-free environment in the Weill Cornell Medicine College vivarium. All animals were randomly assigned for experiments. Mice with myeloid cell-specific deletion of MYC (MYC^ΔM^) were produced by breeding MYC flox/flox mice with LysM-Cre mice as described previously [16]. NRF2 global knock-out (KO) mice were obtained from the Jackson Laboratory (Bar Harbor, ME, USA) and crossed with LysM-Cre mice to obtain experimental NRF2 KO mice on the LysM-Cre background (NRF2 KO). MYC^ΔM^ and NRF2 double knock-out mice (DKO) were generated by crossing NRF2 KO and MYC^ΔM^ mice. Littermate LysM-Cre mice were used as controls (WT).

### 2.2. Mouse Osteoclastogenesis

Medullary cavities of femurs and tibia from mice were flushed with phosphate-buffered saline (PBS) from Corning Inc. (Corning, NY, USA) to harvest bone marrow cells, which were then suspended in ammonium chloride potassium (ACK) lysis buffer from Invitrogen (Waltham, MA, USA) to remove red blood cells. The remaining cells were cultured for one day in alpha-MEM (Invitrogen) media with 10% fetal bovine serum (FBS) from Atlanta Biologicals (Atlanta, GA, USA), 1% l-glutamine (200 mM; Invitrogen), 1% penicillin-streptomycin (5000 U/mL, Invitrogen), and 5% L929 cell supernatant, which provided M-CSF [33]. Then, non-adherent cells were transferred to a new petri dish and cultured for an additional three days. Afterward, the adherent cells were defined as osteoclast precursor cells (OCPs) and used for consequent experiments. For osteoclastogenesis assays, OCPs were plated at a density of 1 × 10^4^ cells per well in triplicates on a 96-well tissue culture plate. The media was exchanged every 2 days until cells were fixed. The plates were fixed and stained for tartrate-resistant acid phosphatase (TRAP) with an Acid Phosphatase Leukocyte Diagnostic Kit from Sigma-Aldrich (St. Louis, MO, USA) following the manufacture’s protocol. For whole-cell protein lysate, OCPs were plated at 1 × 10^5^ cells per well in a 24-well tissue culture plate. For nuclear-protein isolation, 1 × 10^6^ cells were plated in 60 mm tissue culture dishes.

### 2.3. RNA Isolation and Quantitative-PCR

For RNA isolation, OCPs were plated at 5 × 10^5^ cells per well in a 6-well tissue culture plate. RNA was obtained following the protocol from the RNeasy Mini Kit from Qiagen (Germantown, MD, USA). Potential contamination with DNA was removed with DNase (Qiagen) treatment, and 0.5 μg of total RNA underwent reverse transcription using the First Strand cDNA Synthesis Kit (Thermo Fisher Scientific, Waltham, MA, USA). The resulting cDNA was used for quantitative-PCR analysis using the iCycler iQ Thermal Cycler and Detection System (Applied Biosystems, Foster City, CA, USA). For mouse experiments, the expressions of target genes were normalized to that of hypoxanthine-guanine phosphoribosyl transferase (Hprt). The primer sequences for the quantitative RT-qPCR reactions are listed in Supplementary Table S1.

### 2.4. Chromatin Immunoprecipitation (ChIP) Assay

ChIP assay was performed as previously [17]. To describe briefly, 1 × 10^7^ mouse OCPs were fixed directly in the medium using formaldehyde with a final concentration of 1% for 5 min and quenched with glycine. Afterward, cells were harvested, and nuclear contents were isolated. Chromatin was sheared using sonication to a length of approximately 500 base pairs using a Bioruptor sonicator from Diagenode (Denville, NJ, USA). Sheared chromatin was precleared and immunoprecipitated with the NRF2 antibody from Abcam (Cambridge, MA, USA; ab62352) or PBS for negative control. Immune complexes were then washed, and crosslinking was reverted via overnight incubation at 65 °C. Excess proteins were digested with proteinase K treatment for two hours at 55 °C, and then DNA was isolated using a PCR purification kit (Qiagen). Quantitative-PCR was performed as described above in duplicates to detect the binding of NRF2 to specific regions of the genome targeted by primers. ChIP-qPCR results were calculated using the percent input method. Primer sequences are listed in Supplementary Table S1.

### 2.5. Reagents

Human RANKL was obtained from Peprotech (Rocky Hill, NJ, USA). Antibodies (1:1000) used for immunoblotting are as follows: NFATc1 (Santa Cruz, Dallas, TX, USA; 7A6); c-Myc and Lamin B (Abcam; ab32072 and ab16048); a-tubulin (Sigma-Aldrich; T9026); Phospho-c-Myc, NRF2, ERK1/2, p-ERK1/2, IκBα, p-JNK and p-p38 (Cell Signaling Technology, Danvers, MA, USA; 13748, 12721, 9102, 9101, 9242, 9251, and 9215). CDDO-Im, U0126, SP600125, Ly294002, and SB203580 were purchased from Tocris (Minneapolis, MN, USA; 4737, 1144, 1496, 1130, and 1202). *N*-acetyl-l-cysteine (NAC) and actinomycin D were purchased from Sigma-Aldrich (A9165 and A1410).

### 2.6. Bone In Vivo Phenotype Analysis

For studying the in vivo phenotype using micro-computed tomography (μCT) and histomorphometric analyses, the femurs of 12- to 13-week-old female mice were fixed in 4% paraformaldehyde for 3 days after they were obtained. μ-CT analysis [34] was performed as described previously [35], and all samples were included in the analysis conducted in a blinded manner. For μCT analysis, prior to decalcification, femurs with intact joints were scanned using a μCT, with an isotropic voxel resolution of 6 µm (μCT35, Scanco, Brüttisellen, Switzerland; 55 kVp, 145 μA, 600 ms integration time), to evaluate morphological changes in bone. Bone morphology in the femur was examined in two regions: the diaphysis and the metaphysis. For cortical bone, the volume of interest (VOI) encompassed cortical bone within a 231-slice section in the diaphysis. For trabecular bone, the VOI encompassed a 200-slice section in the metaphysis, proximal to the growth plate. To ensure the exclusion of primary spongiosa in the growth plate, VOIs began 50 slices proximal to the median of the growth plate. Outcome parameters for cortical bone included cortical bone volume fraction (BV/TV) and porosity. Trabecular bone parameters included bone volume fraction (BV/TV), trabecular thickness (Tb. Th), trabecular separation (Tb. Sp), and trabecular number (Tb. N). Three-dimensional reconstructions were generated by stacking thresholded 2D images from the contoured region.

After they were analyzed with μCT as described previously [16], femurs were decalcified with 10% buffered EDTA (Sigma-Aldrich) and embedded in paraffin. Sections were cut at a thickness of 7 μm, transferred to a positively charged slide (Thermo Fisher Scientific), and stained with TRAP and methyl green (Vector Laboratories, Burlingame, CA, USA). Then, the histomorphometric slides were analyzed using OsteoMeasure software (OsteoMetrics, Decatur, GA, USA). The histomorphometry experiment was performed with tarsal bones of WT, NRF2 KO, or DKO mice. Bone histomorphometric analysis was performed using a computerized semi-automated system (OsteoMeasure) with light microscopy. The tarsal bones were fixed in 4% paraformaldehyde for 3 days, were decalcified with 10% neutral buffered EDTA (Sigma-Aldrich), and were embedded in paraffin. The quantification of osteoclast was performed in paraffin-embedded tissues that were stained for TRAP and methyl green (Vector Laboratories). Osteoclast cells were identified as multinucleated TRAP-positive cells adjacent to bone. The measurement terminology and units used for histomorphometric analysis were those recommended by the Nomenclature Committee of the American Society for Bone and Mineral Research (Washington, DC, USA) [36].

### 2.7. K/BxN Serum Transfer Arthritis Mouse Model

Arthritis was induced in mice using K/BxN serum prepared as described previously [37]. Briefly, 8- to 9-week-old male WT, NRF2 KO, and DKO mice were given an intraperitoneal injection of 50 μL of K/BxN serum on day 0 and 100 μL on day 2 to induce arthritis. The development of arthritis was monitored for 15 days by measuring the thickness of wrist and ankle joints daily using dial-type calipers (Bel-Art Products, Wayne, NY, USA). For each animal, the joint thickness was calculated as a sum of the measurement of both wrists and both ankles. Joint thickness was represented as an average for each group. The severity of arthritis was scored in a blinded fashion by three researchers for each paw on a 3-point scale: 0 = normal appearance, 1 = localized edema or erythema over one surface of the paw, 2-edema or erythema involving more than one surface of the paw, and 3 = marked edema or erythema involving the whole paw. The scores of all four paws were added for a composite score [38].

### 2.8. RNA Interference

Mouse OCPs were seeded at a density of 3 × 10^5^ cells per well in 6-well plates or 5 × 10^5^ cells in 60 mm tissue culture dishes and transfected with 50 nmol of siRNA oligonucleotides (ON-TARGETplus Non-targeting Pool, D-001810-10-20; SMARTpool: ON-TARGETplus Mouse Nfe2l2 siRNA, L-040766-00-0010, Dharmacon, Lafayette, CO, USA) using TransIT-TKO^®^ transfection reagent (Mirus Bio, Madison, WI, USA) according to the manufacturer’s instructions. Cells were used for experiments after 48 h of incubation.

Primary human CD14^+^ monocytes were purified from buffy coats purchased from the New York Blood Center (New York, NY, USA) by CD14 microbead positive selection (Miltenyi, Auburn, CA, USA) as previously described [16] using a protocol approved by the Hospital for Special Surgery Institutional Review Board (2019-0681, approved 26 April 2019). 0.4 nmol of siRNAs oligonucleotides (ON-TARGETplus Non-targeting Pool, D-001810-10-20; SMARTpool: ON-TARGETplus Human NFE2L2 siRNA, L-003755-00-0020, Dharmacon) were transfected into primary human CD14^+^ monocytes with an Amaxa Nucleofector device using a human monocyte nucleofector kit from Lonza (Basel, Switzerland), as previously described [39]. Cells were cultured at 37 °C, 5% CO_2_ in alpha-MEM medium supplemented with 10% heat-inactivated defined FBS (HyClone Fisher, Logan, UT, USA), l-glutamine, and 20 ng/mL human M-CSF from Peprotech. Cells were used for experiments after 72 h of incubation.

### 2.9. Serum CTX Assay

The serum level of C-terminal telopeptide of type I collagen (CTX-I) from fasted mice was measured using a RatLaps (CTX-I) EIA Kit from Immunodiagnostic Systems (Boldon, UK) according to the manufacturer’s instructions.

### 2.10. Statistical Analysis and Graphs

All statistical tests were performed with GraphPad Prism 8 (San Diego, CA, USA) using one-way ANOVA with Tukey’s post *t*-test for multiple comparisons, two-way ANOVA with Tukey’s multiple comparison test for multiple comparisons with two independent variables, or a two-tailed unpaired *t*-test for two conditions. Shapiro‒Wilk normality tests were performed, and for data that fell within Gaussian distribution, we performed appropriate parametric statistical tests. For those that did not fall within equal variance Gaussian distribution, we performed appropriate non-parametric statistical tests. *p* < 0.05 was taken as statistically significant. Sample sizes were chosen according to standard guidelines. The number of animals was indicated as “*n*.” The Schematic diagram was created using BioRender (San Francisco, CA, USA).

## 3. Results

### 3.1. ERK and p38 Activation Is Required for MYC Expression in Osteoclastogenesis

**Figure 1:**
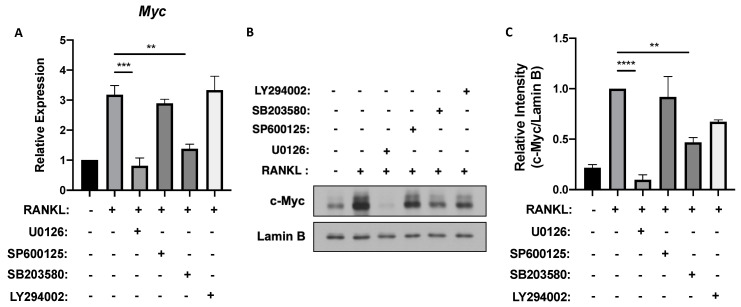
Extracellular signal-regulated protein kinase (ERK) and p38 activations are crucial for MYC expression after receptor activator of nuclear factor-κB ligand (RANKL) stimulation. Mouse osteoclast precursor cells (OCPs) were pretreated with either DMSO (vehicle), U0126 (5 μM), SP600125 (5 μM), SB203580 (10 μM) or LY294002 (5 μM) for 30 min and then stimulated with RANKL (50 ng/mL) for 6 h. (**A**) The mRNA expression of Myc (relative to the hypoxanthine guanine phosphoribosyl transferase (Hprt) housekeeping gene, *n* = 3). (**B**) Immunoblot of nuclear protein lysates using c-Myc and Lamin B antibodies. Lamin B served as the loading control. Data are representative of three experiments. (**C**) Signal intensity of the c-Myc immunoblot in B quantified using densitometry and normalized to Lamin B and to vehicle-treated RANKL control (*n* > 3). All data are shown as mean ± s.e.m. ** *p* < 0.01, *** *p* < 0.001 and **** *p* < 0.0001 using one-way ANOVA in (**A**,**C**); NS, not significant in (**C**).

MYC, a critical regulator of osteoclastogenesis, is primarily induced by RANKL, peaking between 6 to 24 h after RANKL stimulation [16]. To find the upstream regulator of RANKL-induced MYC expression in osteoclastogenesis, we first tested which downstream signaling pathways of RANKL impact on MYC expression. RANKL activates important downstream signaling pathways including MAPK in osteoclast precursors (OCPs) [6]. To block ERK, c-Jun *N*-terminal kinase (JNK), p38, or PI3K/AKT signaling, mouse OCPs were pre-treated with small molecule inhibitors and then stimulated with RANKL. While the treatment with SP600125 (JNK inhibitor) and LY294002 (PI3K/AKT inhibitor) had a comparable expression of MYC mRNA to the vehicle control treatment, inhibiting ERK and p38 activation using U0126 and SB203580, respectively, significantly decreased the mRNA expression of MYC (Figure 1A). Consistently, RANKL-induced MYC protein level was suppressed by U0126 and SB203580 treatments while inhibiting JNK and PI3K/AKT had minimal effects on MYC expression (Figure 1B,C). Inhibiting ERK signaling, in particular, caused the most significant decrease in both transcriptional and translational levels of MYC (Figure 1). As the role of reactive oxygen species (ROS) as a signaling moderator that activates MAPKs such as ERK and p38 has been previously demonstrated [32], we tested if ROS could also affect MYC expression. Treating mouse OCPs with *N*-acetylcysteine (NAC), a ROS inhibitor, decreased both mRNA and protein expressions of MYC (Supplementary Figure S1A,B). Overall, our results suggest that MYC expression is regulated by RANKL-induced ROS, ERK, and p38 pathways.

### 3.2. NRF2 Deficiency Enhances MYC Expression by Promoting ERK and p38 Phosphorylation

**Figure 2:**
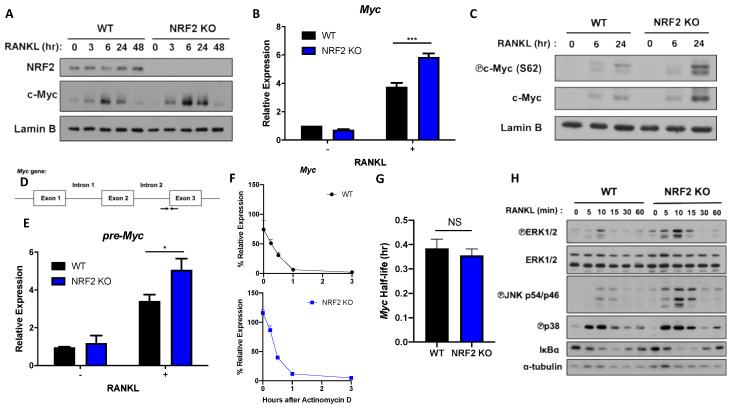
Nuclear factor erythroid 2-related factor (NRF2) deficiency increases MYC transcription and protein expression. Control (wild-type (WT)) and NRF2-deficient (NRF2 knock-out (KO)) OCPs were stimulated with RANKL (50 ng/mL) for the indicated time points. (**A**) Immunoblot of nuclear protein lysates using NRF2, c-Myc, and Lamin B antibodies. Lamin B served as the loading control. Data are representative of three experiments. (**B**) The mRNA expression of Myc (relative to the Hprt housekeeping gene) after 6 h of RANKL stimulation (*n* = 3). (**C**) Immunoblot of nuclear protein lysates using phosphorylated c-Myc (S62), c-Myc, and Lamin B antibodies. Lamin B served as the loading control. Data are representative of three experiments. (**D**) Schematic diagram showing the primers (indicated by the black arrows) designed to detect un-spliced, premature Myc mRNA (pre-Myc). (**E**) Expression of pre-Myc after 6 h of RANKL stimulation (*n* = 6). (**F**,**G**) OCPs were stimulated with RANKL for 6 h and then treated with actinomycin D (10 μg/mL) for 0.25, 0.5, 1, and 3 h. (**F**) Percent expression of Myc after indicated h after actinomycin D treatment (*n* = 3). (**G**) Half-life of Myc transcript in WT and NRF2-deficient OCPs (*n* = 3). (**H**) Immunoblot of total cell protein lysates using p-ERK1/2, ERK1/2, p-JNK, p38, IκBα, and α-tubulin antibodies. α-tubulin served as the loading control. Data are representative of three experiments. All data are shown as mean ± s.e.m. * *p* < 0.05 and *** *p* < 0.001 using two-way ANOVA in (**B**,**E**); NS, not significant using two-tailed, unpaired t-test in (**G**).

**Figure 3:**
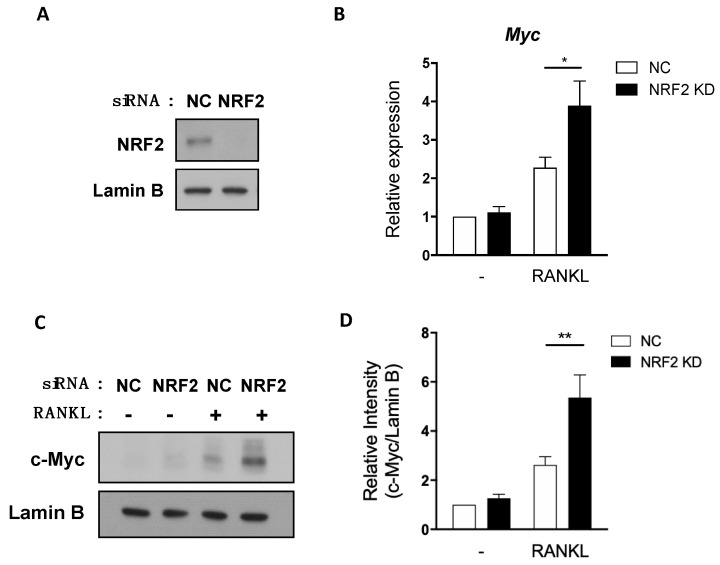
Knockdown of NRF2 enhances RANKL-inducible MYC expression. Mouse OCPs were transfected with negative control (NC) or NRF2-specific small interfering RNAs (siRNAs) and stimulated with RANKL (50 ng/mL) for the indicated time points. (**A**) The expression of NRF2 was determined using immunoblot with nuclear lysates from transfected cells. Lamin B served as a loading control. Data are representative of two experiments from five mice. (**B**) The mRNA expression of Myc (relative to the Hprt housekeeping gene) at 19 h following RANKL stimulation (*n* = 5). (**C**) The expression of MYC using immunoblot with nuclear lysates at 24 h following RANKL stimulation. Lamin B served as a loading control. Data are representative of two experiments from five mice. (**D**) Signal intensity of the c-Myc immunoblot in C quantified using densitometry and normalized to Lamin B and to unstimulated NC control (*n* = 5). All data are shown as mean ± s.e.m. * *p* < 0.05 and ** *p* < 0.01 using two-way ANOVA.

Next, we sought a regulator that lies upstream of the ROS-ERK/p38 pathway and regulates MYC expression. NRF2 is an emerging suppressor of osteoclastogenesis that regulates the intracellular level of ROS by activating transcriptions of various antioxidant proteins such as heme oxygenase-1 (HO-1) and glutathione [23,24,25,27,40,41]. Furthermore, ROS activates NRF2, and NRF2 can suppress osteoclast differentiation by inhibiting the phosphorylation of proximal signaling proteins such as ERK and JNK [25]. Therefore, we tested if NRF2 could regulate MYC. Consistent with the previous reports [24,25,27,40,41], osteoclastogenesis was accelerated in NRF2-deficient OCPs compared to WT OCPs (Supplementary Figure S1C). To determine whether NRF2 regulates MYC expression during osteoclastogenesis, we isolated OCPs from WT and NRF2-deficient mice and measured the MYC mRNA and protein levels upon RANKL stimulation. Both protein and mRNA expressions of MYC were higher in NRF2-deficient OCPs compared to those of WT OCPs (Figure 2A,B), suggesting an inhibitory role of NRF2 in MYC expression. To further understand the NRF2-mediated MYC regulation, we checked if NRF2 affects MYC phosphorylation to stabilize MYC protein [42]. Indeed, NRF2 deficiency increased the level of phosphorylated MYC (Figure 2C), suggesting that NRF2 deficiency regulated the expression of MYC partly via stabilizing MYC protein. We next tested whether NRF2 influences MYC transcription or mRNA stability. To target nascent MYC mRNA before splicing, we designed primers that bind to the second intron and the third exon of the pre-myc mRNA transcript, respectively (Figure 2D). The expression of MYC pre-mRNA in NRF2-deficient cells was higher than that of WT cells (Figure 2E). In addition, we tested whether NRF2 regulates MYC mRNA stability. WT and NRF2-deficient OCPs were treated with actinomycin D when MYC expression was peaked at 6 h after RANKL stimulation. NRF2-deficient OCPs initially had a higher percentage of MYC expression compared to WT after RANKL stimulation (Figure 2F). However, the half-life of MYC mRNA in NRF2-deficient cells was comparable to that of WT, suggesting that NRF2 downregulates MYC expression by targeting MYC transcription, but not mRNA stability (Figure 2G). To gain insight into underlying mechanisms of NRF2-mediated MYC regulation, we tested if NRF2 regulates MYC by modulating RANKL signals. We isolated OCPs from WT and NRF2-deficient mice and measured the activation of the proximal RANKL signaling pathways in WT and NRF2-deficient OCPs. NRF2-deficient OCPs show higher phosphorylated levels of ERK1/2, JNK, and p38 than WT OCPs (Figure 2H). In contrast, the activation of NF-κB remained comparable between WT and NRF2-deficient OCPs (Figure 2G). To further link between enhanced RANKL-induced signaling pathways and increased expression of MYC in NRF2-deficient cells, we treated NRF2-deficienct OCPs with small molecule inhibitors which block ERK, JNK, p38, and PI3K/AKT signaling. Among them, U0126 and SB203580 treatment inhibited the mRNA expression of MYC in NRF2-deficient cells, suggesting that NRF2 suppressed MYC via ERK and p38 pathways (Supplemental Figure S1D). To examine the autonomous role of NRF2 in regulating MYC expression, NRF2 expression was knocked down using small interfering RNAs (siRNAs) directed against NRF2 mRNA, and cells were stimulated with RANKL. NRF2 was diminished by NRF2 knockdown (KD) (Figure 3A). The knockdown of NRF2 significantly decreased both MYC mRNA and protein expressions in mouse OCPs (Figure 3B,C). Similarly, NRF2 KD in primary human monocytes was performed using siRNAs against NRF2 as previously described [39] and decreased the level of MYC mRNA (Supplementary Figure S2A,B). Taken together, our results suggest that NRF2 is an upstream regulator of MYC and negatively regulates MYC transcription in a cell-autonomous manner.

### 3.3. Hyperactivation of NRF2 Suppresses MYC Expression

**Figure 4:**
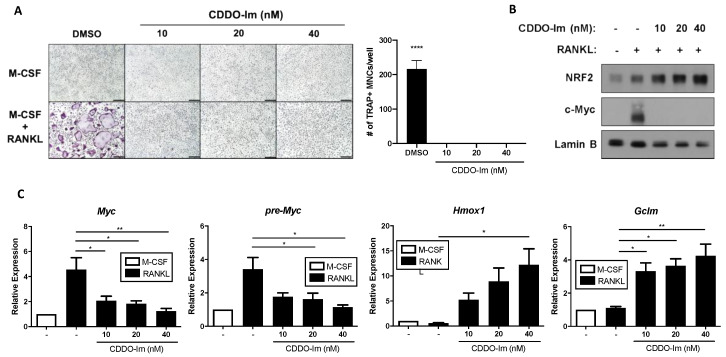
CDDO-Im (1-[2-cyano-3-,12-dioxooleana-1,9(11)-dien-28-oyl] imidazole) treatment suppresses MYC expression and osteoclast differentiation. Mouse OCPs were pre-treated with either DMSO or the indicated doses of CDDO-Im for 30 min and then stimulated with RANKL (50 ng/mL). (**A**) Osteoclast differentiation of OCPs in the presence of DMSO or the indicated doses of CDDO-Im. Representative images of the TRAP-stained cells are shown. Scale bar: 50 μm. TRAP-positive, multinucleated (more than three nuclei) cells were counted in triplicates from three experiments. (**B**) Immunoblot of nuclear protein lysates using NRF2, c-Myc, and Lamin B antibodies after 24 h of RANKL stimulation. Lamin B served as the loading control. Data are representative of three experiments. (**C**) The mRNA expression of Myc, pre-Myc, Hmox1, and Gclm (relative to the Hprt housekeeping gene) after 6 h of RANKL stimulation (*n* = 3). All data are shown as mean ± s.e.m. * *p* < 0.05, ** *p* < 0.01, and **** *p* < 0.0001 using one-way ANOVA in (**A**,**C**).

To corroborate our findings showing that NRF2 negatively regulates MYC expression, we pharmacologically activated the NRF2 pathway in WT OCPs using 1-[2-cyano-3-,12-dioxooleana-1,9(11)-dien-28-oyl] imidazole (CDDO-Im), an NRF2 activator [43,44]. CDDO-Im treatment completely inhibited the formation of multinucleated tartrate-resistant acid phosphatase-positive (TRAP-positive) osteoclasts as well (Figure 4A). CDDO-Im treatment dose-dependently increased the protein levels of NRF2 while simultaneously inhibiting RANKL-induced MYC protein expression (Figure 4B). While CDDO-Im enhanced the expression of well-known NRF2 target genes including Hmox1, a gene encoding HO-1, and Gclm, a gene encoding glutamate-cysteine ligase modifier subunit, both mRNA and pre-mRNA expressions of MYC significantly decreased with the treatment of CDDO-Im in a dose-dependent manner (Figure 4C). NRF2 activation by CDDO-Im treatment also decreased the phosphorylation of ERK1/2, JNK, and p38, which was promoted by NRF2 deficiency (Supplementary Figure S3A).

NRF2 regulates MYC transcription (Figure 2 and Figure 3). Since NRF2 is a well-known transcriptional factor for regulating gene expression including antioxidant enzymes [45], we tested the possibility that NRF2 could directly regulate MYC expression by binding to its promoter. We performed chromatin-immunoprecipitation (ChIP)-qPCR using an NRF2 antibody to assess NRF2 binding to the promoter of MYC. To maximize NRF2 expression, cells were treated with CDDO-Im to induce the expression of NRF2 in WT OCPs either with or without RANKL. We identified two potential NRF2 binding sites (termed R1 and R2) in the upstream region of the MYC promoter and exon 1 of the gene based on the publicly available NRF2 ChIP-sequencing data and potential RNA polymerase II/III-binding sites in the promoter region [46,47] (Supplementary Figure S3B). As expected, we found increased NRF2 enrichment to the promoter regions of Hmox1 and sulfiredoxin-1 (Srxn1), well-known NRF2 target genes, when cells were treated with CDDO-Im (Supplementary Figure S3B). However, we could not detect any significant binding of NRF2 to the upstream region of the MYC promoter (Supplementary Figure S3C), suggesting that NRF2 mediates MYC expression not by direct binding to its promoter region. Taken together, our results using both loss-of-function and gain-of-function models suggest that NRF2 negatively regulates the transcription of MYC by modifying RANKL signaling pathways but not by direct binding to the promoter of MYC.

### 3.4. MYC Is Essential for NRF2 Deficiency-Induced Osteoclastogenesis

**Figure 5:**
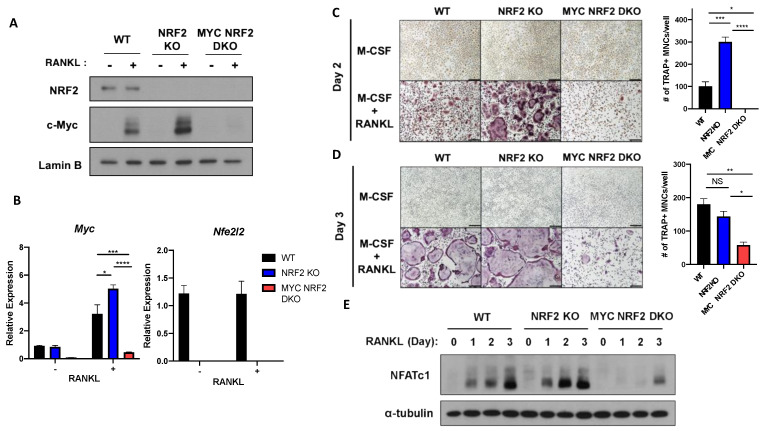
MYC is required for enhanced osteoclastogenesis by NRF2 deficiency. Control (WT), NRF2-deficient (NRF2 KO), and MYC/NRF2-deficient (DKO) OCPs were stimulated with RANKL (50 ng/mL). (**A**) Immunoblot of nuclear protein lysates using NRF2, c-Myc, and Lamin B antibodies. Lamin B served as the loading control. Data are representative of three experiments. (**B**) Expressions of Myc and Nfe2l2 (relative to the Hprt housekeeping gene) after 6 h of RANKL stimulation (*n* = 3). (**C**,**D**) Osteoclast differentiation in WT, NRF2 KO, and MYC/NRF2 DKO OCPs. OCPs were cultured in the presence of RANKL for (**C**) 2 days or (**D**) 3 days, after which they were fixed and stained for TRAP. Representative images of the TRAP-stained cells are shown. Scale bar: 50 μm. TRAP-positive, multinucleated (more than three nuclei) cells were counted in triplicates from three experiments. (**E**) Immunoblot of nuclear protein lysates of WT, NRF2 KO, and MYC/NRF2 DKO OCPs stimulated with RANKL for the indicated time points using nuclear factor of activated T cells (NFATc1) and α-tubulin antibodies. α-Tubulin served as a loading control. Data are representative of three experiments. All data are shown as mean ± s.e.m. * *p* < 0.05, ** *p* < 0.01, *** *p* < 0.001, and **** *p* < 0.0001 using two-way ANOVA in (**A**) and one-way ANOVA in (**C**,**D**); NS, not significant.

To test whether MYC is required for the increased osteoclastogenesis by NRF2 deficiency, we generated NRF2-deficient MYC conditionally-deficient double knock-out mice (MYC^ΔM^/NRF2KO, DKO) by crossing globally NRF2-deficient mice with conditional MYC KO mice (myeloid-cell specific deficiency, Myc flox/flox LysM-Cre mice as described previously [16]). NRF2 and/or MYC expression was efficiently diminished in the knock-out OCPs (Figure 5A,B). To examine the functionality of their osteoclast formation, OCPs from WT, NRF2 KO, and DKO mice were cultured in the presence of M-CSF and RANKL for two days for early time point and three days for later time point of osteoclastogenesis. Mature and multinucleated osteoclasts were formed from NRF2-deficient OCPs on day 2 after RANKL stimulation when WT OCPs stayed as mononuclear preosteoclasts (Figure 5C). At that time, DKO cells failed to form osteoclasts (Figure 5C), suggesting that the early induction and enhancement of osteoclastogenesis by NRF2 deficiency is mediated by MYC. Even after WT cells fully formed multinucleated osteoclasts on day 3, DKO cells only formed a moderate number of osteoclasts (Figure 5D). NFATc1 is a target of MYC [16] and was induced by RANKL stimulation. NFATc1 expression in NRF2-deficient cells was higher on day 1 and 2 compared to WT cells (Figure 5E). DKO cells showed decreased RANKL-induced NFATc1 protein expression compared to those of both WT and NRF2-deficient cells (Figure 5E). These results support that MYC is crucial for enhanced osteoclastogenesis by NRF2 deficiency and NRF2 is an upstream regulator of MYC.

### 3.5. Myeloid-Specific Deletion of MYC Attenuates Osteoclast-Mediated Bone Loss Induced by NRF2 Deficiency

**Figure 6:**
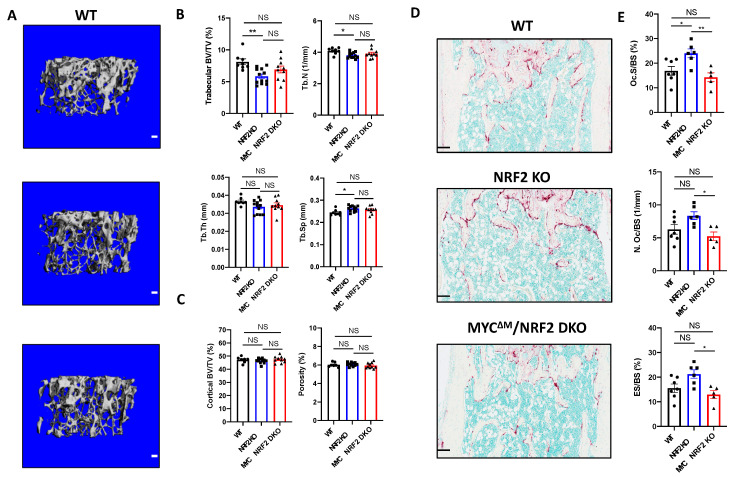
Myeloid-specific deletion of MYC decreases the osteoclast enhancing effect of NRF2 deficiency in vivo. (**A**–**C**) Micro-computed tomography (μCT) analysis of 12- to 13-week-old female WT, NRF2-deficient (NRF2 KO), and myeloid-specific MYC/NRF2-deficient (MYC^ΔM^/NRF2 DKO) mice. (**A**) Representative μCT reconstructed images of the trabecular architecture of the distal femurs. Scale bar: 100 μm. (**B**) μCT measurements of the indicated parameters of the trabecular bone in the distal femurs. Bone volume/tissue volume ratio (BV/TV), trabecular numbers (Tb.N), trabecular thickness, (Tb.Th), and trabecular space (Tb.Sp) were computed using μCT analysis. (**C**) μCT measurements of the indicated parameters of the cortical bone in the midshaft of the femurs. BV/TV and porosity were computed using μCT analysis. Data are shown as mean mean ± standard deviation of at least seven mice per group. (**D**,**E**) Histomorphometric analysis of the trabecular bone in the distal femurs from 12- to 13-week-old female WT, NRF2 KO, and MYC^ΔM^/NRF2 DKO mice. (**D**) Representative images showing the TRAP-positive, multinucleated osteoclasts (red-purple) in the coronal sections of the distal femur. Scale bar: 100 μm. (**E**) Histomorphometric analysis of the trabecular bone. Osteoclast surface area per bone surface (Oc.S/BS). Osteoclast number per bone surface (Oc.N/BS). Erosion over bone surface (ES/BS). All data are shown as mean ± s.e.m. of at least five mice per group. * *p* < 0.05 and ** *p* < 0.01 using one-way ANOVA in (**B**,**E**) except Tb.Th, which was analyzed using Kruskal‒Wallis test; NS, not significant.

We wished to test if the NRF2-MYC axis affects in vivo osteoclastogenesis. Twelve-week-old WT, NRF2 KO, and MYC^ΔM^/NRF2 DKO mice were subjected to micro-computed tomography (μCT) and histomorphometry analysis. In μCT analysis, NRF2 KO mice exhibited significantly lower trabecular bone volume over total volume (BV/TV) and trabecular number (Tb. N) and significantly higher trabecular space (Tb. Sp) than WT mice, while DKO mice showed the trend toward to increasing bone mass compared to NRF2 KO mice (Figure 6A,B). Meanwhile, trabecular thickness (Tb. Th), cortical BV/TV, and porosity were comparable among the groups (Figure 6B,C). Histomorphometric analysis revealed that the osteoclast surface per bone surface (Oc. S/BS) was significantly higher in NRF2 KO mice compared to both WT and DKO mice (Figure 6D,E). In addition, DKO mice exhibited a significantly decreased number of osteoclasts per bone surface (N.Oc/BS), eroded surface area over bone surface (ES/BS), and osteoclast surface per bone surface (Oc.S/B.S) compared to NRF2 KO mice, indicating that increased osteoclast number and function in vivo induced by NRF2 deficiency were reversed by myeloid-specific MYC deficiency. As NRF2 is globally deleted in NRF2 KO mice, we also investigated whether the diminished bone mass in NRF2 KO mice results from defects in the function of NRF2-deficient osteoblasts. Although there were no significant differences in the bone formation rate (BFR) nor mineral apposition rate (MAR) among WT, NRF2 KO, and DKO mice (Supplementary Figure S4A–C), we also found that overt phenotypes including body weight, spleen weight, and femur length were comparable between NRF2 KO and DKO mice (Supplementary Figure S4D–F). However, serum CTX-I was comparable among all groups (Supplementary Figure S4G). Overall, our findings suggest that MYC is required for enhanced osteoclast formation and function in NRF2-deficient mice although deficiency of MYC in myeloid cells was not sufficient to reverse the bone phenotype of NRF2-deficient mice.

### 3.6. Myeloid-Specific MYC Deficiency Alleviates the Bone Loss in Serum Transfer-Induced Inflammatory Arthritis in NRF2-Deficient Mice

**Figure 7:**
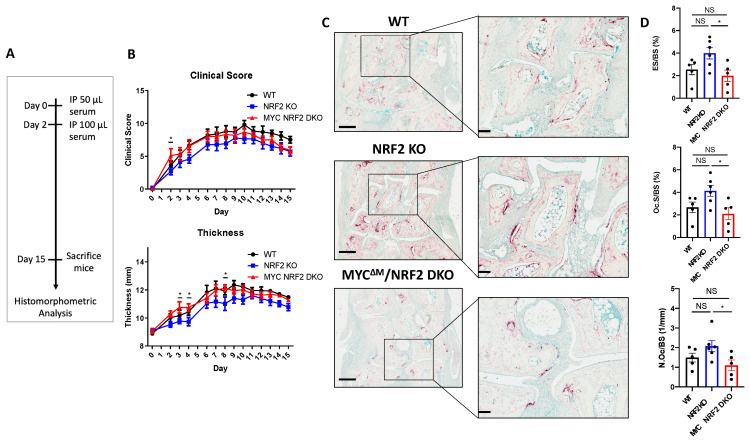
Myeloid-specific deletion of MYC mitigates in vivo osteoclast formation and bone erosion of NRF2-deficient mice in mouse arthritis model. Arthritis was induced by the K/BxN serum transfer method in 8- to 9-week-old male WT, NRF2-deficient (NRF2 KO), myeloid-specific MYC/NRF2-deficient (MYC^ΔM^/NRF2 DKO) mice. (**A**) Schematic timeline of the experiment design. The arthritis-inducing serum was injected into the mice intraperitoneally (IP) on day 0 and 2. (**B**) Time course of the clinical score and swelling of joints during the progression of arthritis. Data are shown as mean ± s.e.m. of at least five mice per group. * *p* < 0.05 between NRF2 KO and MYC^ΔM^/NRF2 DKO groups using two-way ANOVA. (**C**) TRAP staining of histological sections of tarsal bones from WT, NRF2 KO, or MYC^ΔM^/NRF2 DKO arthritic mice. Squares show enlarged images. Left scale bar: 400 μm. Right scale bar: 100 μm. (**D**) Histomorphometric analysis of the tarsal bones. Erosion over bone surface (ES/BS). Osteoclast surface area per bone surface (Oc.S/BS). Osteoclast number per bone surface (Oc.N/BS). Data are shown as mean ± s.e.m of at least five mice per group. * *p* < 0.05 using one-way ANOVA; NS, not significant.

To address the role of NRF2-MYC axis in osteoclast-mediated pathogenic bone erosion, we tested the effect NRF2 and NRF2/MYC double deficiencies on bone loss in K/BXN serum-induced arthritis [48]. K/BxN serum was administrated intra-peritoneally on day 0 and 2, and the severity of arthritis was assessed using a clinical score and ankle joint thickness until day 15 (Figure 7A). There were no significant differences in the joint swelling and inflammation between NRF2-deficient and WT mice (Figure 7B). Conversely, conditional MYC deficiency in myeloid cells restored the decreasing trend of the ankle joint thickness and inflammation in NRF2 KO mice to become comparable to WT mice (Figure 7B), suggesting that MYC could act as a downstream effector molecule of NRF2-mediated inflammatory responses. Histomorphometric analysis of the hind paw tarsal bones revealed an increasing trend of ES/BS, Oc.S/BS, and N.Oc./BS in NRF2 KO mice compared to those of WT mice (Figure 7C,D). Strikingly, DKO mice exhibited significantly less ES/BS, Oc.S/BS, and N.Oc./BS compared to NRF2 KO mice, suggesting that MYC deficiency in osteoclasts might mitigate bone erosion caused by osteoclasts in NRF2-deficient mice during inflammatory arthritis (Figure 7C,D). To test if the newly discovered NRF2-MYC axis occurs in patients with rheumatoid arthritis (RA), we measured the expression of NRF2 and MYC in synovial OCPs from RA patients using publicly available data [49]. While MYC expression was significantly higher in RA synovial CD14^+^ cells compared to CD14^+^ cells from healthy controls (HC), NRF2 expression was significantly lower in RA synovial CD14^+^ cells relative to HC CD14^+^ cells (Supplementary Figure S5A,B). These results demonstrate the inverse correlation between MYC and NRF2 as well as diminished NRF2 expression in RA synovial CD14^+^ cells. Taken together, our results suggest that MYC contributes to pathological bone erosion in NRF2-deficient conditions during inflammatory arthritis.

## 4. Discussion

**Figure 8:**
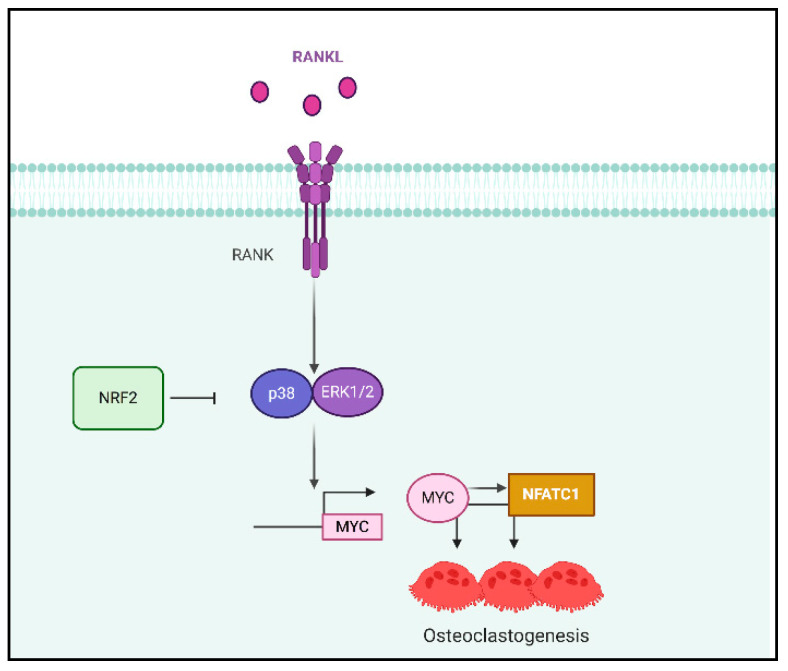
Schematic diagram of the NRF2-regulated mechanism in osteoclastogenesis. NRF2 attenuates the RANKL-induced activation of p38 and ERK-1/2, which is crucial for MYC and NFATc1 expression.

MYC is an important regulator of osteoclastogenesis. However, how MYC is regulated in osteoclasts is not clearly defined. Here we provide the evidence that NRF2 suppresses MYC expression by inhibiting RANKL-induced ERK and p38 activation. We also generated a new line of mice, conditional-MYC /global NRF2 double-knock-out (MYC^ΔM^/NRF2 KO, DKO) mice to elucidate the function of MYC in enhanced osteoclastogenesis by NRF2 deficiency. Knocking out MYC in myeloid cells decreased the enhanced differentiation and NFATc1 expression of osteoclasts in NRF2-deficient cells in vitro. Our study also showed that MYC is a downstream mediator of NRF2-deficient osteoclasts in vivo. Moreover, MYC deficiency contributes to the pathological bone erosion of NRF2-deficient mice in an inflammatory arthritis model. Thus, our data demonstrate the new pathway to regulate MYC expression and to intervene in physiological and pathological bone erosion (Figure 8).

We revealed the crucial role of ERK and p38 phosphorylation in both protein and mRNA expression of MYC during osteoclastogenesis. Both ERK and p38 play an essential role in osteoclastogenesis [8,28,50,51,52] and ERK1 deficiency in mice decreased osteoclast formation both in vivo and in vitro [53]. In osteoclasts, it has been shown that ERK regulates c-FOS and NFATc1 [54]. However, the downstream effectors of RANKL-induced ERK and p38 are not completely understood. Our study demonstrated MYC as a key downstream effector of RANKL-induced ERK and p38. Overexpression of MYC is a driver for a wide range of tumors [55] and the upstream signals driving aberrant MYC expression have been studied. In tumor cells, inhibiting the Ras/Raf/ERK cascade suppresses the expression of MYC mRNA [56] and increases the MYC protein’s half-life via the phosphorylation of Ser 62 [42]. In addition, p38 inhibition has been shown to decrease MYC expression in endothelin-dependent rat aortic smooth muscle cells [29]. We showed that NRF2 not only regulates the transcription of MYC but also the phosphorylation of MYC at Ser 62, suggesting the NRF2/ERK axis also regulates MYC protein stability in response to RANKL. Furthermore, MYC connects ERK and p38 signals to NFATc1 expression. We have shown that MYC directly induces the expression of NFATc1 [16]. Consistently, NFATc1 increased in NRF2-deficient cells where higher MYC expression was observed. Intriguingly, a very low amount of NFATc1 protein expression was detected at day 3 of RANKL stimulation in DKO mice. Since NRF2 deficiency boosted the expression of MYC and the LysM-Cre system did not provide complete deletion of MYC, low levels of MYC expression may have remained and been boosted by NRF2 deficiency in DKO cells. In addition, it is still possible that NRF2 deficiency regulates NFATc1 in a MYC-independent manner. Further studies should be conducted to dissect exactly how phosphorylated ERK and p38 contribute to the transcription of MYC in osteoclasts. Along with ERK and p38, JNK phosphorylation was also found to be elevated upon RANKL stimulation by NRF2 deficiency. Interestingly, inhibition of JNK had no effect on the regulation of Myc mRNA in both the WT and NRF2-deficient OCPs, while the protein expression of MYC was minimally regulated in WT OCPs. There has been a report showing that JNK regulates MYC protein stability in mice fibroblasts [57]. Although our data indicate that JNK may not play a crucial role in the expression of Myc mRNA in osteoclasts, further research could aim to elucidate the potential role of JNK in its protein stability. Taken together, our study provides new insights into the underlying mechanisms of the RANKL-induced ERK and p38 program in osteoclasts.

NRF2 is a negative regulator of osteoclastogenesis. It has been suggested that the negative role of NRF2 in osteoclast formation is mediated by upregulating antioxidant enzymes and degrading ROS [25,40]. However, the mechanisms by which NRF2 suppresses osteoclast differentiation have not been completely explored. Our study established NRF2 is an upstream regulator of MYC. Osteoclast differentiation needs to be balanced to avoid the overly active osteoclasts that lead to excessive bone erosion and cause pathological bone diseases. Our study suggests that NRF2 activation may provide an activation threshold to prevent excessive activation of osteoclasts by controlling the activation of proximal RANKL signaling pathways and MYC expression. In addition, inhibiting ERK and p38 activation using U0126 and SB203580 in NRF2-deficient cells also decreased the expression of MYC to the basal levels. Overall, our study supported that NRF2 is an upstream regulator of the ERK/p38-MYC cascade in osteoclasts and uncovered a new mechanism by which NRF2 regulates osteoclastogenesis.

A wealth of information about the role of NRF2 in biology has been generated based on the studies using NRF2-deficient mice and/or activators of NRF2. In contrast, the effects of global NRF2 deficiency in the bone phenotype remains controversial while the effect of NRF2 on in vitro osteoclastogenesis is consistent across the publication [24,25,27,40,41]. Kim et al. show that 8-week-old female NRF2-deficient mice had lower bone volume than WT mice due to decreased osteoblast formation at around 3-week-old [41]. Another study reports that 9-week-old NRF2-deficient mice had increased bone volume due to increased bone formation rate [27]. Both studies reported no significant differences in the osteoclast number. Meanwhile, 17-week-old male NRF2-deficient mice had decreased bone volume compared to control as a result of a lower bone formation rate and osteoclast number in the trabecular bone [23]. In our study, 12-week-old female NRF2-deficient mice showed significantly less bone volume with higher osteoclast surface per bone surface. The differences in the bone phenotype and the osteoclast activity of NRF2-deficient mice across these studies could have arisen from age, the genetic background, or the different housing environment. Notably, MYC deficiency in osteoclasts significantly decreased osteoclasts in DKO mice, suggesting that increased osteoclastogenesis in NRF2-deficient cells is dependent on MYC. However, it did not fully restore bone density in vivo as indicated by μ-CT analysis, suggesting that other different factors play a role in the actual bone homeostasis. It has been previously reported that NRF2 deficiency interferes with the early bone requisition in mice by inhibiting osteoblast formation [41]. Although we did not see any significant differences in in vivo osteoblast activity among the experimental groups, both NRF2 KO and DKO mice showed a decreasing trend of in vivo osteoblast activity compared to WT mice. This mild reduction in osteoblast activity regulated by NRF2 deficiency may contribute to the discrepancy between the bone parameter and osteoclast parameter and cause the lack of significant change in the DKO bone mass. It is also possible the unknown effect of NRF2 deficiency in other cells or interaction between MYC and NRF2 in cells beyond osteoclasts and osteoblasts could modulate the bone phenotype. Overall, the incomplete rescue of the bone phenotype by MYC deficiency in the DKO mice emphasizes that global NRF2 deficiency and lack of both MYC and NRF2 may regulate bone homeostasis by affecting multiple cell types crucial for proper bone homeostasis.

The NRF2-MYC axis plays an important role in pathological bone erosion. MYC conditional deficiency significantly reduced osteoclast number in NRF2 KO mice in a K/BXN serum transfer-induced arthritis model. The clinical scores and thickness of the NRF2-deficient mice are comparable to those of the control, which is different from a previous report showing a higher arthritic score in the NRF2-deficient mice as a result of greater inflammation [48]. Our study used NRF2-deficient mice carrying the LysM-Cre transgene of a younger age that may exhibit a less inflammatory phenotype than older animals. It has been shown that NRF2 may directly inhibit the transcription of inflammatory cytokines and indirectly limit the inflammatory responses by regulating HO-1 expression [45]. NRF2-deficient mice show augmented activation of proinflammatory genes in the lung after lipopolysaccharide (LPS) challenges [58] and persistent inflammation in the skin of wound healing models [59]. However, although NRF2 is capable of binding to the promoter of proinflammatory cytokines, proinflammatory cytokine production via LPS stimulation in NRF2-deficient mice is comparable to WT mice [45]. However, we have not observed increased inflammation in a murine model of inflammatory arthritis, suggesting the differences in the background and age of the mice used could have led to different severities of arthritic symptoms. Further research needs to be conducted to decipher the effect of NRF2 deficiency on inflammatory responses in inflammatory arthritis.

The NRF2-MYC axis has the potential to be targeted therapeutically. NRF2 inducers such as dimethyl fumarate (DMF) and CDDO-Im are in clinical trials for neurodegenerative and chronic kidney diseases, respectively [60,61]. Oxidative stress induced by reactive oxygen species (ROS) is considered an important contributor for pathological bone erosion although the exact mechanism is not clear. ROS increases with aging and/or estrogen deficiency in postmenopausal women [62]. A significant decrease in plasma antioxidants is also observed in postmenopausal women and patients with rheumatoid arthritis (RA). Treating WT OCPs with CDDO-Im, an NRF2 activator, suppressed the expression of MYC and, concomitantly, decreased the formation of osteoclasts. [63,64]. We also showed that the NRF2 mRNA level was lower in synovial CD14^+^ cells from RA patients, suggesting the potential link between low NRF2 and an increase in osteoclasts and bone erosion in RA patients. The inhibitory role of DMF, another NRF2 activator in osteoclastogenesis has been shown in both RANKL- and LPS- induced osteolysis models [61]. Additionally, various KEAP1 deficiency models in which the levels of NRF2 are elevated report increased osteoclastogenesis both in vitro and in vivo [65,66]. Therefore, identifying the downstream effector of NRF2 is important and activating NRF2 in osteoclast precursor cells could be a therapeutic strategy to modulate pathological bone erosion. Our data suggest that MYC is one of the downstream effectors of NRF2. Therefore, the cross-regulation between NRF2 and MYC could be a potential target for pathological bone erosion.

## 5. Conclusions

In summary, our findings highlight the importance of NRF2 in regulating MYC expression in osteoclasts and identify the mechanisms of how MYC is regulated during osteoclastogenesis. Our study also revealed the importance of the NRF-ERK/p38-MYC axis in controlling osteoclastogenesis and pathological bone erosion and a new pathway that can be targeted for therapeutic intervention of osteoclast-mediated bone disease.

## Supplementary Materials

The following are available online at https://www.mdpi.com/2073-4409/9/9/2133/s1, Figures S1–S5 and Table S1. Figure S1: Expression of MYC after treatment with *N*-acetylcysteine (NAC), Figure S2: NRF2 regulates MYC expression in human osteoclastogenesis, Figure S3: NRF2 indirectly regulates MYC transcription, Figure S4: Myeloid-specific MYC/NRF2-deficient mice exhibit decreased mineral apposition rate (MAR), Figure S5: The inverse relationship between MYC and NRF2 in synovial CD14^+^ macrophages isolated from rheumatoid arthritis, Table S1: The list of primers used in the study.
