# Supplementary material for: NRF2 Is an Upstream Regulator of MYC-Mediated Osteoclastogenesis and Pathological Bone Erosion
Source: Cells. 2020 Sep 21;9(9):2133. doi: 10.3390/cells9092133 (PMC7564846; doi:10.3390/cells9092133)
Supplement: Supplementary file 1 [file cells-09-02133-s001.pdf]

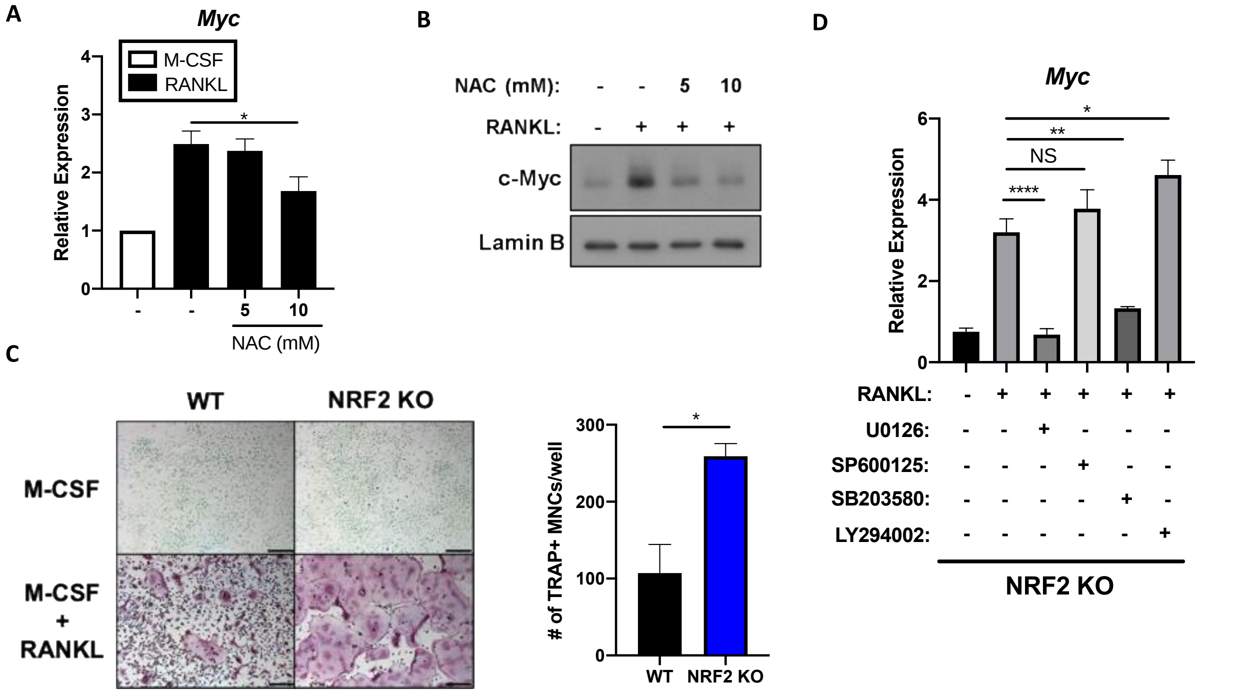


**Supplementary Figure 1.** Expression of MYC after treatment with N-acetylcysteine (NAC). (**A,B**) Mouse OCPs were pre-treated with the indicated concentration of N-acetylcysteine (NAC) for 30 minutes and then stimulated with RANKL (50 ng/ml) for 6 hours. (**A**) The mRNA expression of *Myc* (relative to the *Hprt* housekeeping gene, *n*=5). (**B**) Immunoblot of nuclear protein lysates using c-MYC and Lamin B antibodies. Lamin B served as the loading control. Data are representative of five experiments. (**C**) Osteoclast differentiation of WT and NRF2-deficient (NRF2 KO) OCPs after stimulation with RANKL (50 ng/ml) for 2 days. Representative images of the TRAP stained cells are shown. Scale bar: 50 µm. TRAP-positive, multinucleated (more than three nuclei) cells were counted in triplicates from four experiments. (**D**) NRF2-deficient OCPs were pretreated with either DMSO (vehicle), U0126 (5 µM), SP600125 (5 µM), SB203580 (10 µM) or LY294002 (5 µM) for 30 minutes and then stimulated with RANKL (50 ng/ml) for 6 hours. The mRNA expression of *Myc* (relative to the *Hprt* housekeeping gene, *n*=3). All data are shown as mean ± s.e.m. **P* < 0.05, ***P* < 0.01, and *****P* < 0.0001 by one-way ANOVA in **A,D** and by two-tailed, unpaired t-test in **C;** NS, not significant.

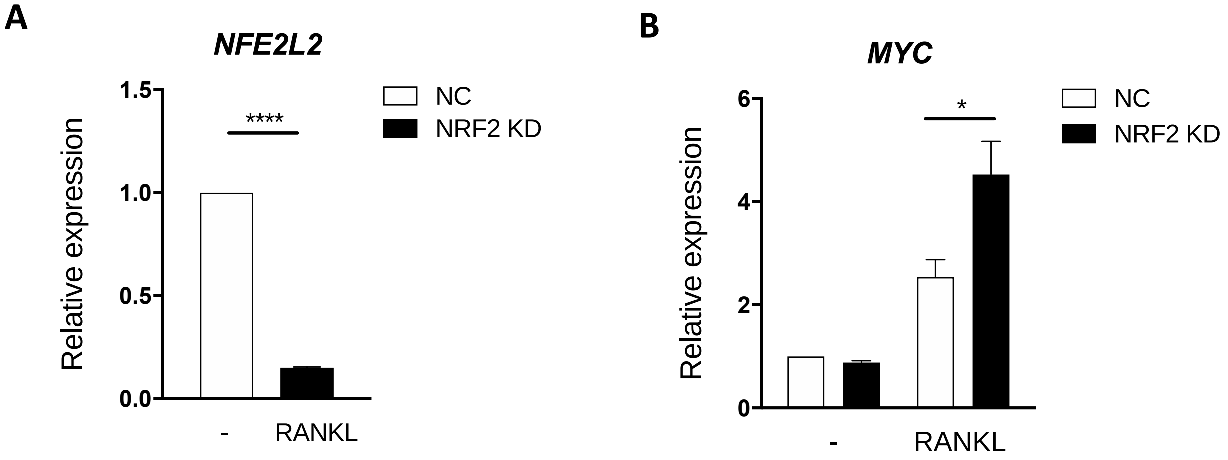


**Supplementary Figure 2.** NRF2 regulates MYC expression in human osteoclastogenesis. Primary human monocytes were nucleofected with negative control (NC) or NRF2-specific small interfering RNAs (siRNAs) and then stimulated with RANKL (40 ng/ml). (**A**) The mRNA expression of *NRF2* (*NFE2L*, relative to the *HPRT* housekeeping gene) after transfection ($n$=4). (**B**) The mRNA expression of *MYC* (relative to the *HPRT* housekeeping gene) at 9 hours following RANKL stimulation ($n$=4). Data are shown as mean ± s.e.m. from three independent experiments with four independent donors. **** $P < 0.001$ by two-tailed, unpaired t-test in **A**; * $P < 0.05$ by two-way ANOVA in **B**.

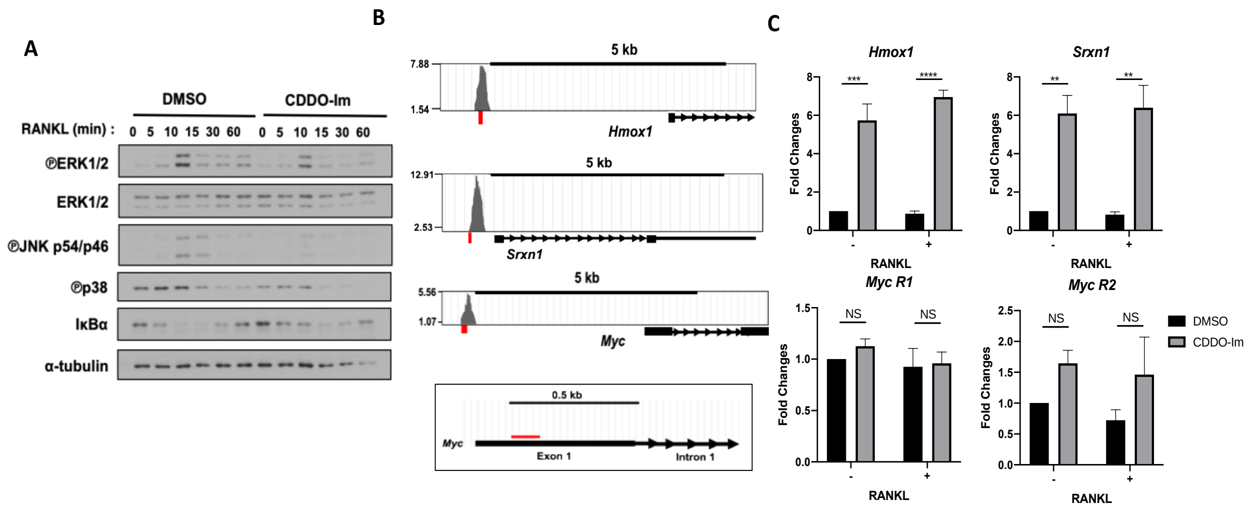


**Supplementary Figure 3.** NRF2 indirectly regulates MYC transcription. (**A**) Immunoblot of total cell protein lysates using p-ERK1/2, ERK1/2, p-JNK, p-p38, IκB$\alpha$, and $\alpha$-tubulin antibodies. 20 nM of CDDO-Im and 100 ng/ml of RANKL for the indicated times were used for this experiment. $\alpha$-tubulin served as the loading control. Data are representative of three experiments. (**B,C**) Mouse OCPs were pretreated with DMSO or CDDO-Im (20 nM) for 30 minutes and then stimulated with RANKL (50 ng/ml) for 6 hours. Afterwards, cells were fixed and processed for ChIP (chromatin immunoprecipitation). (**B**) ChIP-seq track near the promoter region of *Hmox1, Srxn1* and *Myc* genes as well as the first exon of *Myc* on the UCSC genome browser based on publicly available data. Red rectangles indicate where the designed ChIP-qPCR primers bind to. (**C**) ChIP qPCR analysis of potential NRF2 binding sites for *Hmox1, Srxn1* and *Myc* gene promoter regions (normalized to *Hbb-b1* gene). Myc binding primers targets the NRF2 binding region upstream of the Myc gene while the myc promoter primers bind to the first exon of MYC where it contains the potential RNA polymerase III promoter sequence ($n$=3). All data are shown as mean ± s.e.m. **$P < 0.01$,*** $P < 0.001$, and **** $P < 0.0001$ by two-way ANOVA; NS, not significant.

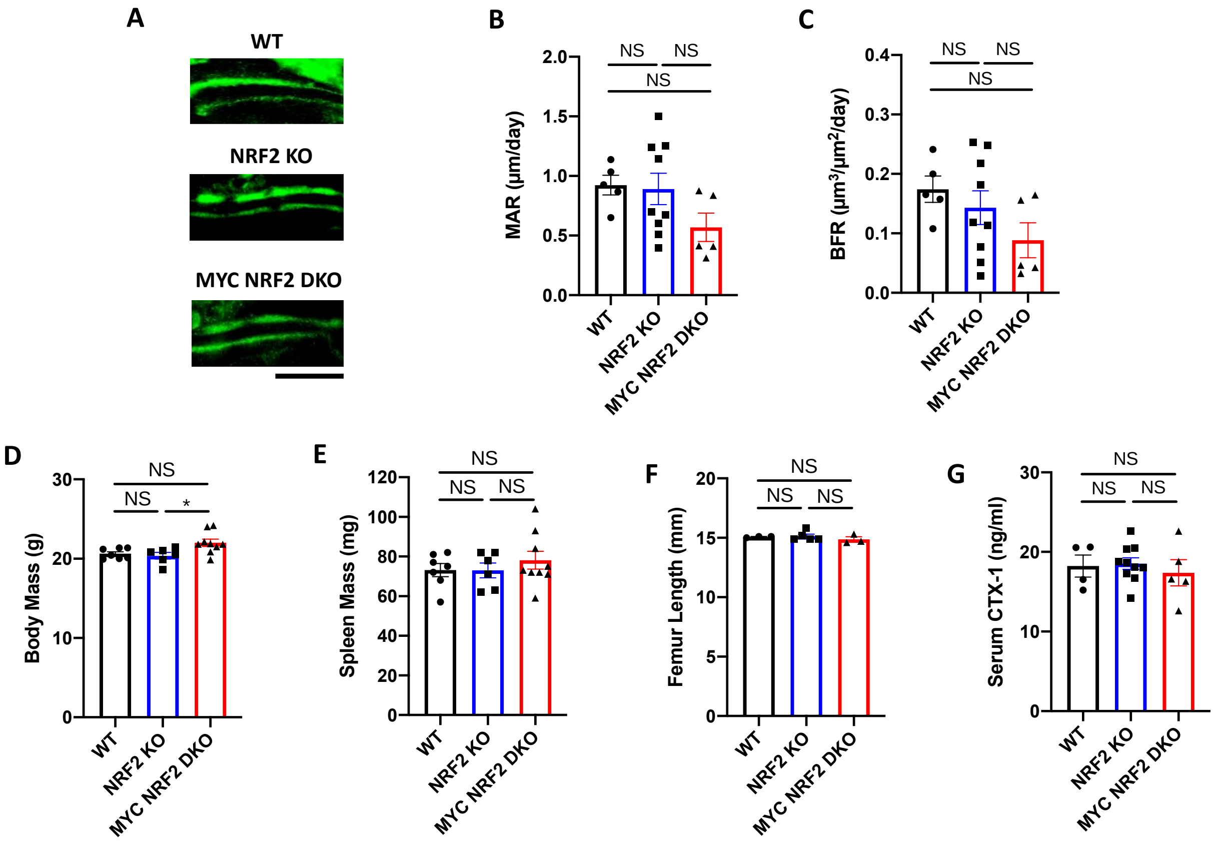


**Supplementary Figure 4.** Myeloid-specific MYC/NRF2-deficient mice exhibit decreased mineral apposition rate (MAR). **(A,B,C)** Bone formation parameters of 12 to 13 weeks old female WT, NRF2-deficient (NRF2 KO), and myeloid-specific MYC/NRF2-deficient ($MYC^{\Delta M}$/NRF2 DKO) mice (n ≥ 5). (**A**) Representative images of calcein double labeling in the trabecular bone. Scale bars: 10 µm. Bone formation parameters such as (**B**) mineral apposition rate (MAR) and (**C**) bone formation rate (BFR). (**D,E,F,G**) Body mass (**D**), spleen mass (**E**), femur length (**F**), and serum level of CTX-1 (**G**) of 12 to 13 weeks old female WT, NRF2 KO, and $MYC^{\Delta M}$/NRF2 DKO mice. Data are shown as mean ± s.e.m. of at least four mice per group. *$P < 0.05$; NS, not significant by one-way ANOVA in **B,D,E,G** and by Kruskal-Wallis test in **C,F**.

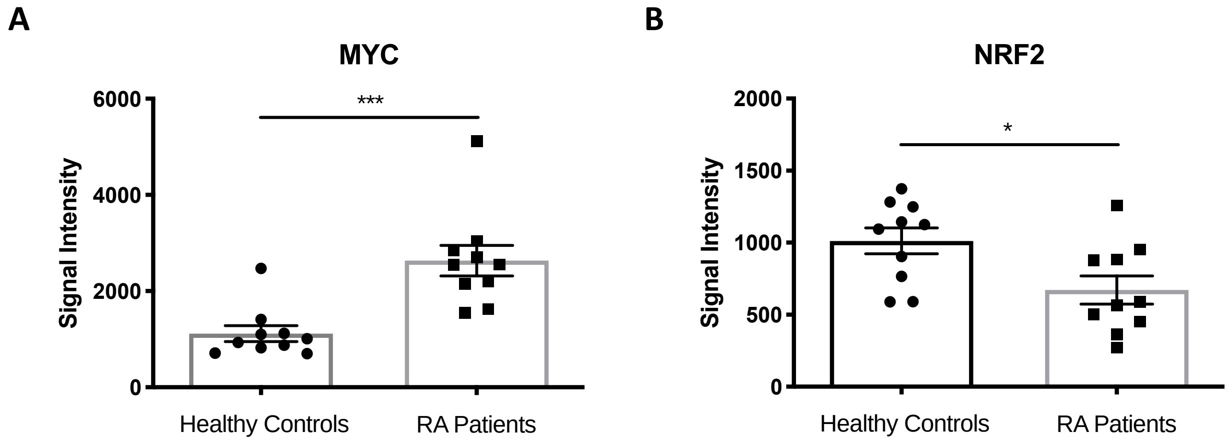


**Supplementary Figure 5**. The inverse relationship between MYC and NRF2 in synovial $CD14^+$ macrophages isolated from rheumatoid arthritis**. (A,B)** Comparisons of NRF2 and MYC expressions in synovial $CD14^+$ macrophages isolated from the joints of healthy controls or patients with rheumatoid arthritis (RA). Publicly available dataset (GEO: GSE97779) was used for the data ($n$=10). All data are shown as mean ± s.e.m. ***$P < 0.001$ by two-tailed, Mann Whitney test in **A** and *$P < 0.05$ by two-tailed, unpaired t-test in **B.**

**Supplementary Table 1**. The list of primers used in the study.

| Gene Symbol | Quantitative PCR Primer Sequence |
|---|---|
| *MYC* | F: 5'-GTGCATCGACCCCTCGGTGG-3'<br>R: 5'-TTGCGAGGCGCAGGACTTGG-3' |
| *NFLE2L2* | F: 5'-GTCCCAGCAGGACATGGAT-3'<br>R: 5'- CGTCGCTGACTGAAGTCAAAT -3' |
| *HPRT* | F: 5'-GACCAGTCAACAGGGGACAT-3'<br>R: 5'-CCTGACCAAGGAAAGCAAAG-3' |
| *Myc* | F: 5'-GCCGATCAGCTGGAGATGA-3'<br>R: 5'-GTCGTCAGGATCGCAGATGAAG-3' |
| *Nfle2l2* | F: 5'-TGAAGCTCAGCTCGCATTGA-3'<br>R: 5'-TGCTCCAGCTCGACAATGTT-3' |
| *Hmox1* | F: 5'-GAGCAGAACCAGCCTGAACT-3'<br>R: 5'-AAATCCTGGGGCATGCTGTC-3' |
| *Gclm* | F: 5'-GACAAAACACAGTTGGAACAGC-3'<br>R: 5'-CAGTCAAATCTGGTGGCATC-3' |
| *Pre-Myc* | F: 5'-TGTGATCTTCCACTTCCTCCCT-3'<br>R: 5'-GACCTCTTGGCAGGGGTTTG-3' |
| *Hprt* | F: 5'-TCCTCAGACCGCTTTTTGCC-3'<br>R: 5'-CTAATCACGACGCTGGGACT-3' |
| **Gene Symbol** | **ChIP quantitative PCR Primer Sequence** |
| *Hmox1* | F: 5'-CCCCACAGGAGCTGAACTTT-3'<br>R: 5'-TCTGCTAATCACCCCTCCCA-3' |
| *Srxn1* | F: 5'-TGGCTTTACTTCGTGGAGGC-3'<br>R: 5'-AGATCTGCCCAGAGAGGATGA-3' |
| *Myc R1* | F: 5'-GTAGCTCAGAGACAAAGCCC-3'<br>R: 5'-TCCTGTGCCACTCTACCTAC-3' |
| *Myc R2* | F: 5'-TGGCGGGAAAAAGAAGGGAG-3'<br>R: 5'-CCCTCTGTCTCTCGCTGGAA-3' |
| *Hbb-b1* | F: 5'-TGCTCAGAATCAAACCCAAGG-3'<br>R: 5'-GGGCAACAATGATTTGGGTGC-3' |
